# Skeletal dysplasias: approach to the clinical diagnosis and implication of appropriate diagnosis for knowledge and research studies in these rare diseases. Hereditary multiple Osteochondromas as example/paradigm

**DOI:** 10.1186/1824-7288-40-S1-A8

**Published:** 2014-08-11

**Authors:** Maria Gnoli, Elena Pedrini, Marina Mordenti, Morena Tremosini, Luca Sangiorgi

**Affiliations:** 1 Department of Medical Genetics and Skeletal Rare Diseases, Rizzoli Orthopaedic Institute, Bologna, Italy

## 

The skeletal dysplasias are a large, heterogeneous group of genetic conditions characterized by abnormal development, growth and maintenance of the elements (bones) comprised in the human skeleton [1]. In the 2010 revision of nosology and classification of genetic skeletal disorders, 456 conditions were included and placed in 40 groups defined by molecular, biochemical, and/or radiographic criteria. Of these conditions, 316 were associated with mutations in one or more of 226 different genes [2], and are present in about 5% of children with birth defects [3]. About 100 skeletal dysplasias have prenatal onset [4] with ultrasound findings particularly in the second trimester [5]. The first step for an accurate diagnosis is a detailed clinical-radiographic evaluation [4]. In fact, because of clinical and genetic heterogeneity of these diseases, with partial clinical overlap, diagnosis is difficult with a consequent delay in specific follow-up and management.

Clinical and molecular characterization of a large patients series is the first step that leads to an improvement in knowledge about natural history, epidemiology and pathogenesis of this disease. These advancements are promoted by expertise centres where patients can be followed-up by multidisciplinary teams, required for syndromic nature or different skeletal segments involvement in the most of cases. To improve skeletal disease knowledge, often lacking, it is essential to analyze and integrate available clinical and genetic data; to this purpose, the design and development of disease-specific registries [5] is essential, as well as the presence of a Biobank for the collection of biospecimens.

Our experience as Reference Centre for Skeletal Dysplasias led us to activate specific diseases registries, as Multiple Osteochondromas Registry (REM), using an HL7 compliant platform, GePhCARD (Genotype-Phenotype Correlation, Analyses and Research Database) that can encompass clinical, genetic, genealogical and imaging data [6]. This web-application is protected by an authentication system, a relief tool articulated in multilevel access profile for data legal protection and patients’ privacy. GePhCARD will be soon interfaced with BIOGEN (**Gen**etic **Bio**bank) and together will contribute to improve diagnosis and clinical and molecular characterization of rare diseases, allowing to collect high quality biological materials of skeletal dysplasias patients [7].

In this presentation we focus on our experience on a specific skeletal disease, Hereditary Multiple Osteochondromas (MO), and demonstrate how a systematic integration of clinical and molecular data is focal to increase the knowledge on MO natural history and epidemiology [8], contributing also to define personalized and appropriate follow-up and to hypothesize research studies.
